# Important but challenging: Chinese in-service EFL teachers’ research attitudes in a graduate program

**DOI:** 10.3389/fpsyg.2023.1142496

**Published:** 2023-08-24

**Authors:** Lei Chen, Quanguo Liu

**Affiliations:** ^1^School of International Studies, Shaanxi Normal University, Xi'an, China; ^2^School of Foreign Languages, Lanzhou University of Finance and Economics, Lanzhou, Gansu, China

**Keywords:** research attitudes, teacher research, mixed methods, research methodology, in-service teacher development, reasoned action approach

## Abstract

In recent decades, the practice of involving teachers in research in degree programs is becoming popular. Yet, little is known about the impact of research experiences on teachers’ behavior: whether research experiences change their teaching practices and lead to further research efforts in future careers, especially in the unique social, cultural, and educational culture of China. Thus, this study examines Chinese IETs’ (in-service EFL teachers’) research attitudes in a graduate program with a reasoned action approach. We used an embedded mixed methods research design to investigate a cohort of 197 IETs who completed the Research Attitudes in Vocational Education Questionnaire (RAVE-Q). The quantitative data validates the survey and shows that, in general, the IETs hold positive attitudes toward research. Next, semi-structured interviews investigating IETs and their advisors’ perceptions of teacher research were conducted. The qualitative data shows a variety of research experiences among the IETs. Specifically, this study highlighted some interviewed IETs who had the desire to be reflective about their teaching and to apply research in their practices, nevertheless, the educational contexts made such efforts impossible. Thus, this study questioned the previous assumptions that positive research attitudes lead to corresponding research behaviors. This study offers implications for EFL graduate programs seeking to improve IETs’ research attitudes both within and outside China.

## 1. Introduction

Today, teachers worldwide are increasingly university-educated, and China is no exception (Loughran, 2002). Given the growing trend of degree inflation, many countries have witnessed increasing demand for teachers to have a master’s degree or higher degree to secure a job or a promotion (Bachan, 2017; Byman et al., 2020). To earn a graduate degree, pre-and in-service teachers are often required to conduct original research projects related to teaching practices (China’s Ministry of Education, 2007; Eklund, 2019).

However, researchers disagree in terms of the impact of such research on teachers. Teacher research might empower teachers (Cochran-Smith and Lytle, 2009) and bring enhanced professional knowledge and understanding of research, as well as personal growth (Dobber et al., 2012; Yarullin et al., 2015; Admiraal et al., 2017; Aspfors and Eklund, 2017; Eklund, 2019). Other researchers find that the practicality of teacher research is vague (Westbroek et al., 2022), not useful, and sometimes disconnected from the practice (Yancovic-Allen, 2018). Thus, the role of research in teacher education programs remains unclear (Aspfors and Eklund, 2017). Similarly, little evidence exists on the research experiences and attitudes of English teachers although English teachers are increasingly required to conduct research on their practices whether or not they enroll in degree programs (Wong, 2014; Yancovic-Allen, 2018; Byman et al., 2020).

The current study examines IETs who are full-time in-service teachers with a bachelor’s degree in education. After they enrolled in a graduate education program, they were trained and required to examine their teaching through research inquiries, as a result the following questions arise: (1) What factors characterize the research attitudes of IETs enrolled in a graduate program in China? (2) What factors influence their attitudes toward research during their research experiences? (3) What factors influence their willingness to conduct empirical research in their future profession and change their teaching practices accordingly?

This study aims to investigate the research attitudes of IETs enrolled in a Chinese master’s program with mixed methods. The specific focus is on identifying the research attitudes of these teachers and the challenges they face during their research endeavors. The study administered a survey to the IETs, which was followed by one-on-one interviews with the IETs and some of their advisers in order to contextualize the IETs’ research attitudes within the larger settings of the program. The study has implications for effective guidance, curriculum design, policies, and initiatives that can promote and support teacher research in various contexts.

### 1.1. The unique Chinese social and cultural contexts of in-service teacher research

The Chinese educational context was selected as a unique setting for this study because EFL education has been undergoing revolutionary development in recent decades. China has a long tradition of competitive high-stakes national examinations strongly linked to social status (Yu and Suen, 2005). While this examination-driven system has been widely criticized for its incompatibility with the fast-changing world, it has proven to be quite effective in terms of skill acquisition, considering Shanghai’s successful performance in the Program for International Student Assessment (PISA) exam (Zhao, 2014). Therefore, K-12 teachers in general, and English language teachers, in particular, are caught between struggling to maintain highly performance-oriented and teacher-centered teaching and adapting to student-centered and communicative methods of EFL teaching (Zhang and Liu, 2014; Guo et al., 2016).

China’s most recent curriculum reform policy, which came into effect in 2022, explicitly indicates that teachers are expected to be deeply involved in the learning and research community in which classroom-based action research projects and self-reflection are fostered to promote their professional development. Teachers are encouraged to actively engage in educational research to critically examine and enhance their teaching practices (Du, 2011). However, it is reported that the top-down model of teacher research diminishes rather than promotes EFL teachers’ research initiatives. Chinese secondary English teachers reported pressure to conduct research, yet the support and guidance are insufficient (Gao et al., 2011; Yan and Yang, 2019). Thus, this setting inherently contains tensions and gaps regarding practicing teachers conducting research. Given the large number of Chinese normal universities offering master’s degrees to in-service teachers, there is an urgent need to understand teacher researchers’ research attitudes and practices. Acquiring such knowledge is essential for enriching teacher education programs tailored to in-service teachers, as well as generating valuable insights to enhance educational policies with a focus on cultivating research-oriented teachers who are effective in their practice.

### 1.2. Teacher research in educational degree programs

Teacher inquiry can be explored within two interrelated yet distinct contexts: teachers’ voluntary research aimed at examining their instructional impact, and teacher research conducted to meet the requirements of educational degree programs. Both forms of research involve teachers engaging in systematic inquiry to investigate and reflect upon their teaching practices.

Teachers’ voluntary research practices are typically self-initiated and driven by personal interests or specific classroom challenges. These inquiries often have a flexible timeline and may focus on addressing immediate instructional needs. Early studies highlighted the significance of teacher inquiry (Dewey, 1929) and the promotion of reflectivity in teacher development (Schön, 1983). More recent articles have reported various professional and pedagogical gains resulting from teachers’ inquiry practices. These gains include the improvement of pedagogical knowledge (Gelfuso and Dennis, 2014), the development of research literacy (Evans et al., 2017), the cultivation of an inquiry habit of mind (Kreijns et al., 2019), and increased awareness of cultural premises to create inclusive classrooms (Gay and Kirkland, 2003).

However, it is important to note that teacher research conducted as part of a degree program differs significantly from teachers’ spontaneous research projects, despite the observation of many of the aforementioned benefits in both contexts (Aspfors and Eklund, 2017). In degree programs, teacher research must serve as an assessment requirement for the credential (Kowalczuk-Walędziak et al., 2019), which is subjected to specific guidelines and timelines. However, most previous studies on English teachers’ research experience have either focused on a short period of research experience (Yan, 2017), voluntary research (Gao et al., 2011; Wong, 2014), or pre-service teachers who were not yet fully responsible for teaching while conducting their research (Guilbert et al., 2016; Yan, 2017). There is a need to examine in-service teachers’ attitudes toward mandatory research components in degree programs, especially considering the increasing popularity of such practices worldwide without adequate scrutiny (van der Linden et al., 2015).

### 1.3. Teacher research attitudes

In alignment with recent teacher education discourse, we understand research attitude as a multidimensional continuum and dynamic construct within the reasoned action approach framework. Teachers’ research attitude determines the likelihood of teachers’ research after graduation and is a highly important construct for understanding teacher research experiences (Griffioen, 2019).

The reasoned action approach of Ajzen and Fishbein (2010) provides a strong and widely tested model regarding aspects that affect intentions for individual behavior. According to Ajzen and Fishbein (2010), human social behavior follows reasonably and often spontaneously from people’s information or beliefs about the behavior under consideration. Specifically, three kinds of beliefs are distinguished, and they guide the decision on whether to perform the behavior in question. The first dimension is a positive or negative attitude toward a certain behavior, which describes people’s beliefs about the positive or negative consequences they might experience if they perform the behavior. The second dimension is the perceived norm associated with the behavior, which describes how people form beliefs about whether the important individuals or groups in their lives would (dis) approve of their performing the behavior. Thirdly and finally, people also form beliefs about personal and environmental factors that can aid or impede their attempts to perform the behavior. This is behavioral control perception or the self-efficacy dimension. Contextual conditions such as social conditions and barriers to and facilitators of behavioral performance moderate the effect of intentions on behavior as actual behavioral control.

Based on the reasoned action approach, the Research Attitudes in Vocational Education Questionnaire (RAVE-Q) scale was developed. Several studies have been conducted using the RAVE-Q scale (Blömeke et al., 2015; van der Linden et al., 2015; Griffioen, 2019), all of which evaluated its psychometric properties and reported high levels of internal consistency (Perception of research in profession, *α* = 0.88; Cognitive attitudes, *α* = 0.91; Positive affective attitude, *α* = 0.93; Negative affective attitude, *α* = 0.75; Self-efficacy *α* = 0.86; Importance of Research *α* = 0.87; Intended behavior scale, *α* = 0.94.) (Griffioen, 2019). This study found that undergraduate students’ research efficacy and research intention in future professional practice correlated highly with their perceptions of and attitudes toward research. However, contextual influences on students’ research attitudes were not sufficiently discussed in these studies.

Apart from the RAVE-Q scale, several other theoretical frameworks, namely those proposed by Kowalczuk-Walędziak et al. (2019), van Katwijk et al. (2019), and Vieira et al. (2021), have emerged as potential options. However, these frameworks have yet to undergo testing regarding the psychometric properties of the associated questionnaires.

## 2. Methods

### 2.1. Program contexts

This study explores the research attitudes of teachers enrolled in a master’s teacher education program in China and what contextual factors influenced such decisions. All students enrolled in such a program are full-time in-service teachers who already have an undergraduate degree in teacher education. Within 5 years of completing their undergraduate degree, they can apply to gain entry to the master’s teacher education program. The program includes on-site and online courses and a research project, culminating in writing a master’s thesis. On-site courses are taught during summer breaks. Courses cover disciplinary and pedagogical knowledge and research skills such as searching for and analyzing academic literature, collecting and analyzing classroom data, and thesis writing. Each student in the program is assigned two mentors: a university faculty member (usually tenured or a full professor) and a mentor teacher from the school where the IETs teach.

To fulfill the master’s degree requirements, all teachers need to complete a thesis that involves empirical research on issues that emerged from their teaching. The process of writing this thesis usually spans one to 3 years. Within this timeframe, in-service teachers should move through a process similar to that in other master’s degree programs. This process typically includes proposing a topic, writing a proposal, defending the proposal, writing the thesis, and defending the thesis before a committee. Typically, the committee comprises three members: (1) a professor from the program, (2) a professor from a teacher education program at a different university, and (3) a schoolteacher with teacher research experience and expertise in the same subject matter as the IET. Thus, the research-based thesis component is compulsory for obtaining a master’s degree. However, by incorporating schoolteachers as course instructors, thesis advisors, and committee members, the program also encourages IETs to explore teaching practices.

### 2.2. The mixed-methods research design

This study adopted the embedded mixed methods design (Creswell and Clark, 2011). Before beginning the study, all participants’ written informed consent was obtained for the publication of any potentially identifiable images or data included in this article. Next, the RAVE-Q questionnaire was administered to assess the teachers’ research attitudes. The findings of the quantitative study guided the design of the interviews. Specifically, the quantitative data revealed general experience and opinion patterns, representative of the whole teacher group, while the qualitative study results provided details to clarify the patterns, complexities, and nuances observed in individual cases.

The questionnaire items were based on the RAVE-Q questionnaire in Griffioen (2019), which contains *cognitive* (students’ understanding of the need to conduct research), *affective* (the degree to which students enjoy doing research), *self-efficacy* (levels of self-belief when conducting research), and *research intention* (the likelihood of students conducting research in the future). All items were measured on a 5-point Likert scale, ranging from 1 = *not at all applicable* to 5 = *entirely applicable*. The RAVE-Q questionnaire was translated into Chinese, and the survey questions were tested in a sample of 20 IETs from the 2017 cohort of students enrolled in a master’s teacher education program. The phrasing of some questionnaire items was modified based on the results. The trial stage ensured that the respondents could correctly interpret the translated questionnaire items.

The questionnaire items were modified in two aspects. Firstly, RAVE-Q was designed for all majors in universities. In adapting the RAVE-Q for pre-service teachers in this study, we used more specific vocabulary for the profession. For example, we used “teachers” instead of “professionals,” and “teaching” instead of “professional practices.” Secondly, RAVE-Q was originally designed for college students, however, the participants in our study are already employed. As a result, the phrases were changed from future tense to present tense. For instance, the phrase “I expect I will become a professional who has developed the competencies to do valid research” was adapted to “As a teacher, I have developed the competencies to do valid research.”

Guided by the patterns the quantitative study results revealed, the IET interview questions were designed to gather evidence, details, and cases to inform further exploration of teachers’ experiences and attitudes toward teacher research. The interview protocols centered on the following questions:

1. Did you enjoy conducting research during your graduate program? Which aspects did you enjoy? Which were the least enjoyable?
2. Do your colleagues conduct research? If so, what kinds of research do they conduct?
3. Do you perceive conducting research as important for your career? Why or why not?
4. What do you think is the outcome of your research?
5. Have you noticed any changes in your perception of research since your research experience?
6. Do the people around you, such as your advisors, parents, and colleagues, support your research efforts?
7. Do you feel that you are capable of conducting research? Why or why not?
8. What are the challenges involved in conducting educational research?
9. Are you planning to conduct more research after completing your degree?

We also interviewed the professors/academic advisors who participated in the master’s program to understand their perspectives on teacher research. The interviews with academic advisors centered on the following questions:

Did the IET under your guidance…

Enjoy conducting research in your opinion? Why or why not?
Perceive conducting research as important for their work and career?
Feel that they receive support from other important people in their lives?
Experience any changes in their perception of education/teaching after their research effort?
Demonstrate the ability to conduct research?
Show willingness to conduct more research in the future?
While supervising research…
How do you support your IET? Can you give some examples?
What do you perceive to be the outcome of IET research?
What was challenging for you in terms of advising on IET research?

### 2.3. Quantitative and qualitative data collection

Survey data were collected from participants who graduated from the program within 1 year. The questionnaire was completed by 197 IETs from the 2017 and 2018 cohorts, for a 73% response rate. As the final survey question, we requested participants to provide us with their email addresses if they volunteered to participate the interviews. We contacted the IETs via their email addresses and invited them to face-to-face follow-up interviews. We carefully selected IETs that reflect a diverse range of gender, teaching locations, and study topics. We also asked the IETs whether they would be willing to invite their advisers to participate in the interview study and provide their advisors’ perspectives on the IETs’ research attitudes. We interviewed 16 IETs and eight academic members participating in the program one-on-one. The duration of each interview was 30 min to 1 h. Pseudonyms are used to report the interview results in this study.

Of the 197 IET participants, 21 (11%) were male, and 176 (89%) were female; 74 (38%) teach in cities, 115 (58%) teach in a town, and nine (5%) teach in a rural area. Among the 16 IET interviewees, three IETs were male, and 13 were female; seven teach in a municipality-level city, eight teach in a town, and one teaches in a rural area. The demographic information of the interviewed IETs is demonstrated in Table 1. The faculty interviewees’ demographic information is presented in Table 2.

**Table 1:** Demographic information of the interviewed IETs.

Number	Pseudonyms	Gender	Years of teaching	Contexts of teaching	Research topic
1	Jun	Female	6 years	High school in town	Continuous writing task
2	Li	Female	6 years	High school in the rural area	English learning motivation
3	Wei	Female	7 years	High school in town	Critical thinking and reading
4	Shuang	Male	8 years	Highschool in city	Reading strategies
5	Xue	Male	7 years	Highschool in town	Speaking and assessment
6	Jian	Female	6 year	Highschool in town	Linguistic phoneme
7	Hua	Female	7 years	Elementary school in city	Speaking and technology
8	Liu	Female	6 years	Highschool in city	Classroom discourse analysis
9	Cheng	Female	7 years	Highschool in city	Jigsaw puzzles and reading
10	Lai	Female	6 years	A middle school in town	Peer-editing and writing
11	Yutong	Female	5 years	A middle school in town	Cultural awareness in textbooks
12	Yijun	Female	5 years	Highschool in town	Reading strategies
13	Ruyuan	Female	6 years	Elementary school in a city	Vocabulary teaching
14	Wu	Male	7 years	A middle school in town	Teaching listening and multimodality
15	Guo	Female	6 years	Elementary school in a city	Mind-mapping
16	Wang	Female	8 years	A middle school in town	Reading strategies

**Table 2:** Demographic information of the interviewed faculty members.

Number	Gender	Years of advising in this program	Expertise
1	Male	10 years	Translation studies
2	Female	10 years	Applied linguistics
3	Female	8 years	Learning strategies
4	Male	7 years	Early literacy
5	Female	3 years	Curriculum studies
6	Female	5 year	English literature
7	Male	6 years	American literature
8	Female	5 years	Applied linguistics

The interview participants are volunteers. Volunteers are widely used in interview studies as they tend to be the participants who are articulate, reflective, and willing to share with the interviewer (Morse, 1991). As Table 1 demonstrates, to some extent, the participants are diverse: male and female participants were included; the participants were from a variety of teaching contexts such as rural and urban, primary schools, middle schools, and high schools; the participants’ research topics also varied. Nevertheless, the participants might be the ones who had a higher than average motivation for research considering that they are interested in the topic enough to participate in our research. This limitation is further discussed in the discussion section.

### 2.4. Data analysis

The first data analysis phase outlined the quantitative features of IETs’ research attitudes. Cronbach’s alpha, mean, and standard deviation values were calculated for all seven dimensions of the RAVE-Q. One of the dimensions, **negative affective attitude*,* had a Cronbach’s alpha below 0.60, and when the survey items comprising that dimension were deleted, Cronbach’s alpha for *affective attitude* increased significantly. Hence, the **negative affective attitude* dimension was* deleted from the questionnaire used in this study. The results showed high levels of internal consistency (Perception of research, *α* = 0.82; Cognitive attitudes, *α* = 0.84; Importance of research, *α* = 0.84; Positive affective attitude scale, *α* = 0.81; Self-efficacy, *α* = 0.82, Intended behavior scale, *α* = 0.80.) Next, confirmatory factor analysis (CFA) was performed to validate the six-dimension RAVE-Q in the Chinese IET sample.

The second phase of analysis focused on exploring the qualitative features of IETs’ and their advisors’ perspectives on teacher research. First, the recorded interviews were transcribed and translated. The data were then organized using NVivo 10. Two researchers performed the first round of open coding. In this process, data were analyzed based on the codes that emerged from the interview content. Keywords and *in-vivo* codes were used to summarize the key point in the interview excerpts. Examples of codes at this stage include *understanding of the research-mentor*, *understanding of the research-professional requirement*, *relation to teaching*, *unrelated to teaching*, *motivation for research*, *evaluation of research experiences*, *writing the literature review*, *data analysis*, *difficulties with writing*, *reflection about teaching*, *school contexts*, *peer teachers*, *home support*.

In the second round of qualitative coding, using the constant comparative method (Strauss, 1987), we categorized coding into a few different categories which were also guided by the frameworks of the reasoned action approach (Ajzen and Fishbein, 2010) and the RAVE-Q questionnaire dimensions. For example, IETs’ discussion of their perceptions of the importance of research in relation to their mentors, promotions, and professional requirements, were coded as an *understanding of research influenced by mentor*/promotion/requirements respectively, which were further coded as *the perception of teacher research* (e.g., Research can help solve pedagogical problems). Responses indicating the participants’ evaluation of the importance of research in relation to their personal benefit in the workplace were coded as *evaluation of research* or *motivation for research* in the first round, and then, *cognitive attitudes toward research* for the second round (e.g., Research can help me develop as a teacher). Responses indicating the participants’ positive or negative personal feelings were coded as *positive/negative personal feelings* for the first round and then *Affective attitude toward research* for the second round (e.g., Reading academic literature feels great, as I finally got some input for myself after a day of teaching/output).

Finally, we observed a number of codes that focus on the contextual conditions of research, such as *school contexts*, *school cultures*, *home contexts*, and *home support*, which do not fit into the dimensions of the questionnaire. As a result of this, two researchers performed the third round of analysis and extracted the following common themes:

1. IETs’ self-rating of different facets of their research attitude
2. The relationship between different facets
3. The contextual conditions that influence IETs’ efficacy and research intention

Throughout the coding process, the two researchers coded the data independently for the first and second rounds. After the second round of coding, we compared codes and discussed any discrepancies until we reached an agreement. We also performed the third round of analysis collaboratively to ensure that our analysis meet the requirement for consistency in qualitative research (Armstrong et al., 1997).

## 3. Results

The first research question, which concerns the overall pattern of teachers’ research attitudes, is addressed based on the results of CFA and descriptive analysis of the RAVE-Q questionnaire results, complemented by illustrations from the qualitative findings to support the (included) six of the seven tested dimensions in the construct. The general pattern according to the quantitative finding is also discussed in this section. The second and the third research questions, which concern the contextual conditions that may affect teachers’ research attitudes, are answered based on excerpts from the interviews with the IETs and their advisors, who reported a variety of research experiences. This study finds that successful and rewarding research experiences may be had if there is successful mediation of the institutional requirements for academic research projects. In contrast, when IETs experienced tension between their teaching and research, they tended to regard such experiences as negative. These results raise discussions about the extent to which graduate programs can guide and assist teachers’ professional development.

### 3.1. Underlying dimension of teacher research attitude

This study used Amos 23 to perform CFA to validate the structural validity of the revised RAVE-Q questionnaire for the sample of IETs. CFA is a version of factor analysis used to test and confirm the number of factors and their correspondence with explicitly specified indicators (Brown, 2015). CFA results are reported in Table 2, which shows that teacher research attitude comprises six constructs: perception of research in the teaching profession, cognitive attitude toward research, the importance of research, positive affective attitude toward research, research self-efficacy, and intention to conduct research (Table 3).

**Table 3:** Error and fit index for revised RAVE-Q scale.

Fit index	CMIN/DF	p	GFI	AGFA	CFI	RMR	RMS
Acceptable fit	≤5	>0.05	≥0.9	≥0.9	≥0.9	≤0.10	≤0.08
Model fit	4.862	0.00	0.939	0.907	0.933	0.033	0.046

Results indicated that χ^2^ = 436.3, df = 155, χ^2^/df = 2.814, *p* = 0.000 (GFI = 0.939, AGFI = 0.907, NFI = 0.919, CFI = 0.933); all these incremental indices are scaled from 0 (no fit) to 1 (perfect fit), with 0.95 indicating a good fit. According to the results, almost all indices were close to 0.95. Furthermore, RMSEA = 0.048, and since this value is less than 0.08, it indicates a reasonable error of approximation. According to all the abovementioned CFA results, the model comprises six factors, providing a good model fit for using the RAVE-Q in the Chinese in-service teacher population.

Regarding the sample items, Cronbach’s alpha, mean, and standard deviation values for each dimension are presented in Table 4. The standard deviation ranged from 0.52 to 0.84, and the skew and kurtosis indices were 0.087 and 0.174, respectively. However, these skew and kurtosis indices are unlikely to make any substantive difference to the analysis due to the sample size (Tabachnick et al., 2007). The internal consistency of the confirmed RAVE-Q scale was computed for all given dimensions. The results are indicated in Table 4. The Cronbach’s alpha value shows that the instrument is reliable.

**Table 4:** Six factors of teacher research attitude.

Sample items	Factor loading	Cronbach’s alpha	Mean	SD
Perception of research in the teacher profession		0.82	4.23	0.67
A teacher needs to be aware of the relevant results of research.	0.75			
It is necessary that teachers learn how to conduct research.	0.75			
Teaching can be better validated by research.	0.73			
Teachers develop usable knowledge through research.	0.63			
Cognitive attitude toward research		0.84	4.22	0.84
Gaining knowledge and skills in research is important for my job as a teacher.	0.85			
I need to improve my teaching by conducting research.	0.84			
It is important for me to develop a new understanding of teaching through conducting research.	0.68			
III. Importance of research		0.84	4.25	0.61
Research experience is important for my profession.	0.74			
Research is regarded as important in this program.	0.79			
Professors and college students work on research projects collaboratively in this graduate program.	0.68
IV. Affective attitude toward research (positive)		0.81	3.83	0.56
I find research an exciting way for me to learn something.	0.55			
I find research fascinating because I can discover things myself.	0.68			
I find research fascinating because I can decide on the topic to examine.	0.60			
V. Self-efficacy		0.82	3.86	0.52
I can play an important role in teacher research.	0.63			
I can conduct research that is connected with my teaching.	0.58			
I can employ appropriate research methods to conduct teacher research.	0.75			
I can solve the problems in conducting teacher research.	0.60			
VI. Intention to conduct research in teaching		0.80	3.48	0.75
I will research to improve my teaching after graduation.	0.75			
I will use research to solve the problems of my teaching after graduation.	0.73			
I will conduct research in my future teaching after graduation.	0.57			

Table 5 briefly presents the interview findings related to the six dimensions of teacher research attitude, complemented by excerpts from the interview transcripts.

**Table 5:** Interview excerpts representing each dimension in RAVE-Q.

	Example codes	Interview excerpts that support the statements in RAVE-Q
I. Perception of teacher research	Understanding of research-mentor	My mentor teacher in the school has a graduate degree and she knows about research and theories.
	Understanding of the research-future profession	Teachers are required to conduct research by the school district.
	Understanding of research-teaching	Conducting research might provide empirical evidence about how to improve teaching.
	Unrelated to teaching	In my school, many teachers joke that we are not teachers, we are tiger moms. We do not teach, we just push the students to do the practice, do the practice, and do more practice… so there is nothing to research about.
II. Cognitive attitude toward research	Motivation/reason for research	I wanted to do something with reading because I feel my students have a lot of difficulties with English reading.
	Evaluation of research experience	Some of the research experiences are helpful for me, but sometimes, I spent time fixing the APA format and that is a waste of time.
III Importance of research	Program requirement	Research is a requirement for graduation so everybody around me conducts research in this program.
IV. Affective attitude toward research	Positive feelings	Reading academic literature and talking to my advisor in this program feels so great; I finally get some “input” for myself.
	Positive feelings	As a new teacher, I enjoy having a record of my development, my teaching, and my reflection.
	Negative feelings	Conducting research takes way more time than I had expected.
V. Self-efficacy toward research	Description of “I can”/“I cannot”	We started the project with little understanding of research and a lot of doubt (about how to do research). Now, after many rounds of revision, I am so happy to finally be able to see this thick thesis paper that is written by me.
	Description of “I can”/“I cannot”	After (the thesis) research, I know that if I want to experiment with my students, I can collect data through tests, scales, interviews, and class observations. You need to think about the reliability and validity of your tests. To do research, you have to be thoughtful about the details and rigorous and robust.
	“I cannot”	Sometimes I feel that it is impossible for me to meet my advisor’s requirements.
VI. Research behavior	Reflecting	The literature about “question type” talks about these seven kinds of questions. I am starting to grow this habit of reflecting on what kinds of questions I had asked in classes.
	Future research-positive	I will conduct research in the future. Conducting research is one of the requirements for promotion.
	Future research-negative	I might not conduct this kind of research because it is too time-consuming. But I might read academic literature and search for new ideas for teaching since I know how to read them now.

The interviews’ coding is different from the dimensions in the RAVE-Q in three aspects. First, the excerpts from the interviews are more detailed and contextualized than the statements in the questionnaire. For instance, the item “A teacher needs to be informed of the relevant outcomes of research” is listed in the questionnaire. We discovered that throughout the interview, participants tended not to make such a broad statement. Instead, they supplied concrete examples: “My mentor teacher at the school understands research methods and research theories and has academic publications, thus I feel research is important for promotion in my environment.” This passage was classified as *an understanding of research-professional demand* and *an understanding of research-mentor*. The exemplary codes are provided in Table 5 to reveal how we made connections between the interview excerpt and the factors in the questionnaire.

The second difference between the qualitative and quantitative data is that the negative attitudes and negative feelings are expressed through low scores on positive questionnaire items in the survey. However, in the interviews, the negative sentiments were expressed with negative expressions. For instance, low *self-efficacy* is typically expressed as a low score for “I can” statements in the questionnaire, such as a low rating for “I can solve problems in conducting teacher research.” In interviews, we observed a series of “I cannot” expressions, such as “Sometimes, I feel that it is impossible for me to meet my advisor’s requirements.”

Third, some interview excerpts could be classified into two different factors. For instance, a statement in self-efficacy may also involve a statement about positive or negative “feelings” toward research, which could be classified as *an affective attitude* toward research. An example is “being able to conduct research feels great.” In such cases, we decided that the emphasis of the statement is “being able to” instead of “feelings” and thus, we coded it as *self-efficacy*. The researchers discussed such ambiguous codes in multiple group meetings until we reached consent.

### 3.2. The positive attitudes and the negative experiences

Based on the survey results, the IETs reported a generally positive attitude toward research. Furthermore, most of the interviewed teachers discussed research as meaningful and important for the teaching profession in terms of personal enrichment, enhanced knowledge about scientific research, opportunities to reflect on their practice, and credentials for career advancement. Thus, the qualitative analysis supports the survey results and illustrates them with examples which are discussed in this section.

However, analysis of the interviews also shows diversity in teacher attitudes toward research. Although some teachers had positive attitudes toward research and desired to be reflective, they experienced challenges and disconnection between research and teaching practices. To some extent, they experience frustration exactly because they believed that research could make a change. We discuss the details in the sections below.

#### 3.2.1. Cognitive attitudes toward research

In the reasoned action approach, people’s evaluations of, or attitudes toward the object are determined by their salient or readily accessible belief about the object. This dimension is interpreted in this study as teachers’ cognitive understanding of whether research is important or useful. In the questionnaire, the *mean* for *cognitive attitude toward research* is 4.22 (*SD* = 0.84), which is high. In the interviews, IETs reported that conducting research helped reflect on pedagogical practices and personal assumptions about the students, and for professional promotions. The following excerpts explain their choices. Jun reported that she rated her *perception of research in the teaching profession* so high because her role as a researcher enabled her to examine and expand her pedagogical skills.

My research topic is “continuous writing tasks.” It is a new kind of task in English classes, and I had no experience with it while I was a student … My advisor suggested that I observe some experienced teachers and analyze how they teach. So, this project gives me opportunities to talk to them and ask, “Can I observe your class? I need to write this paper.” […] They offered me suggestions to teach this task which expand my pedagogical skill.

The above excerpt demonstrates that Jun received support for her inquiry in the form of her academic advisor’s guidance and her school’s friendly collegial culture.

Conducting research also gives teachers opportunities to communicate with students in new ways and reflect on their assumptions. The following excerpt captures Li’s experience as a high school teacher in a rural area:

*I interviewed some students during the course of my research. I asked them about their motivation, and I had one student … who was mediocre, in my opinion. She told me that her dream was to go to Paris … She thought Paris was so romantic and eccentric … She drew the Eiffel Tower in her diary. I felt I had underestimated rural students’ motivation for foreign language learning.*

In the following excerpt, Wei discusses her heightened awareness of the use of questions in teaching, as a result of her research on classroom discourse:

*I wanted to do something with reading because I feel my students have a lot of difficulties with English reading. They can't quite grasp the meaning. But my advisor said that is a very broad topic, so I read some previous papers and narrowed it down to: Can critical thinking-oriented class discussion enhance students' reading? […] I started to read and think about critical thinking when I could.*

Conducting research allowed the teacher researchers to reflect on their practices and assumptions. Other than the examples illustrated above, IETs also reported that they found reading academic literature to be enriching because they were “finally [able to] get some input after a day of output.” Two IETs reported that their workplace required that they conduct research, and thus, they found the research training process valuable for their career advancement.

#### 3.2.2. Perception of research in the teaching profession

In the questionnaire, the mean for the *perception of research in the teaching profession* is 4.23 (*SD* = 0.67). All the interviewed IETs reported that they rated five for most of the questions in this dimension. Furthermore, IETs explained their choices as they faced workplace peer pressure to conduct research, as evidenced by the following remark:

*Research experience is important. My mentor teacher has a graduate degree and has been published. The other young teachers who work at my school have graduate degrees. Promotion requirements include research experience and publications.*

IETs also face peer pressure to conduct research and earn a graduate degree from their former undergraduate classmates, as evidenced in the following comment:

*A lot of my former classmates from undergraduate school have graduate degrees, so they can do research. I at least need to do it once so that I know what it is about.*

People generally base their decisions on perceived social pressure to engage in or refrain from performing a behavior. In the case of Chinese EFL graduate IETs, their understanding of the importance of research is affected by their understanding of the norms of conducting research and related peer pressure. IETs typically work in contexts where research in the form of a thesis paper or published journal articles is essential to securing a promotion.

#### 3.2.3. Importance of research in the program

In the questionnaire, the mean for the *Importance of the research* is 4.65 (*SD* = 0.61). All the interviewed IETs reported that they rated 5 for this dimension. Some interviewees chuckled when they explained their rating for this dimension, saying that: “Obviously the whole program centered around research” and “I would not have applied for this program if I did not consider research as important.”

#### 3.2.4. Affective attitudes toward research

The IETs in this study reported the mean for *affective attitude toward research,* 3.83 (*SD* = 0.56). In the interviews, we explored both the positive and negative affective attitudes expressed by the teachers.

In the interviews, IETs reported various reasons that conducting research “felt good,” including the following: completing such a huge task gives them a sense of accomplishment, the experience of reading academic literature can be enriching and enjoyable, and discussing teaching with advisors and peers can be cathartic. Take, for example, the following interview excerpt:

*I quite enjoy going back to the university to take classes and go to the library with my peers. Just walking on campus makes me feel youthful and hopeful again.*

Apart from these descriptions of positive affective attitudes toward research, three interviewed IETs shared a negative affective research experience. They described the research as time-consuming without a worthwhile outcome, as evidenced in the following comment:

*Conducting the research and writing the report was so time-consuming. It was way more effort than I had expected.*

The interviewed IETs also cited logistical issues in their expressions of negative affective attitudes toward research, as evidenced in the following statement:

*I became pregnant three months after I started this graduate program … My mom helped me take care of my baby, but everything is still hard with a baby, [including having] a teaching job and a research [paper] to write.*

On the subject of school support for IETs, one advisor commented as follows:

*I had this one student [an IET], and I felt her school did not support her. I did not know whether it was her making excuses or not. Whenever we arranged to meet, she always said her school required her to do something that she could not put off. I told her, you should talk to your school because otherwise, you won’t graduate, but I felt her school just did not support her. The school wanted to exploit them [IETs].*

Thus, the IETs who participated in this study reported diversified negative affective attitudes toward research, citing various challenges that they faced. They understood the importance of research for their job and for improving teaching but found that successfully conducting research requires considerable time, energy, and support.

#### 3.2.5. Research self-efficacy

The mean for *research *self-efficacy* is* 3.86 (*SD* = 0.52). Bandura (1997) proposed the concept of self-efficacy, which is defined as the personal belief that one is capable of achieving certain goals. If teachers perceive that they can effectively conduct research, their intention to engage in research should be high. In the interviews, among the examples of high research self-efficacy, nine teachers reported that their research experience enhanced their knowledge of scientific research. One such remark is given, as follows:

*After the research, I truly understood concepts like controlled class and experiment class. I started to understand what robust research is. If you want to do a robust intervention study, you have to carefully think about the variables and control a lot of variables. Those are some key ideas for scientific research.*

However, seven interviewed IETs reported that they rated some questions on their self-efficacy as four or three out of five. Reading academic papers is cited as an explanation for their low rating as reading is a daunting task after a tiresome workday. Academic papers require a certain degree of mastery of specific academic skills, in addition to leisure time and energy, which some teachers did not have, as evidenced in the following comments:

*Academic papers are difficult to read. After a tiresome day of teaching, I literally doze off reading them.*

*In the beginning, I wasn't familiar with the reading requirements. I probably used the wrong keywords in the search, so I could only find a few pieces of literature. After I finally read them through, my advisor said these papers are from unqualified journals (not peer-reviewed), and they don't count.*

Despite the program’s honorable intention of providing IETs with the benefits of research-intensive courses and guidance, academic reading for research is undoubtedly hard.

Furthermore, IETs mentioned their struggle with other aspects of conducting research such as collecting and analyzing the data, structuring their report, preparing PowerPoint presentations for the defense, and formatting the report. In the following quote, Xue mentioned her dilemma in deciding the theme of her research. While she valued her idea as it involves novice practices in teaching, the same practice was criticized by the advisor for not being creative enough.

*I had this great idea that I wanted to examine a novel practice in my school--we used a new app for assigning English homework that focus on English language speaking. But my advisor read my work and said, the tasks assigned in the app were mainly for pronunciation and there was a lack of students’ creative uses of language [in the usages of the app]… So now, I need to reconsider everything I did and every word I wrote.*

The following example illustrates the gap between the academic requirements of thesis writing and some IETs’ capacity to meet that requirement:

*Writing the report is very difficult. I have talked to my advisors many times. One gave a lot of advice about focusing on the topic, following the structure (of a thesis paper), etc. She was very patient and gave a lot of advice, but it just felt like her requirements were way beyond me. It was impossible to meet her requirements.*

This feeling of “impossible to meet her requirements” is a typical example of the lack of self-efficacy for research that IETs experienced. To triangulate the IETs’ complaints, we interviewed academic advisors and gathered their opinions about the IETs’ academic reading and writing issues. The advisors explained that they regard academic reading as an essential process in teacher research in terms of cultivating IETs’ critical thinking skills and familiarizing the IETs with the academic conventions of research. In the interviews, the advisors repeatedly complained that the IETs did not read enough, so they lacked adequate reflection and knowledge about research conventions. One such comment is given, as follows:

*These teachers may read one or two papers and feel satisfied with their knowledge of the field. Without enough reading, they [teachers] may only partially understand different methodologies. Their writing tends to be narrow-minded. They saw one interesting idea in one article and took it as a golden rule … I gave them many comments on their writing that asked them to reexamine the reading.*

According to the advisors, by writing and critically responding to the academic literature, IETs can expand their pedagogical knowledge and develop the ability to critically examine different pedagogues. Nevertheless, without providing enough assistance, scaffolding, and guidance in every step of IETs’ research effort, and focusing on requiring IETs to meet the academic standard instead of helping them dissect their teaching, the program witnessed that several interviewed participants reported low self-efficacy in conducting the research.

### 3.3. Intention for future research: being innovative in a high-stakes examination-driven educational system

In the questionnaire, the IETs rated the mean for *intention to conduct research as* 3.48 (*SD* = 0.75). Although this rating is lower than the rating for all the other dimensions, the differences are not significant (*p* > 0.05). Despite that IETs had rated their intention to conduct research in the future high in the questionnaire, all 16 IETs in the interviews, even the IETs who reported that they rated most questions in this dimension five out of five, reported multiple challenges that they had to face in conducting research.

Furthermore, the interviews reveal a diversity in whether such challenges can be reconciled. One industrious IET encapsulated her determination and commitment to future research despite all the difficulties of research, by stating, “No pain, no gain.” Some IETs cited the requirement for teacher research in job place as a reason that they will conduct research in the future, stating that: “Conducting research is a requirement for teachers nowadays.” They detailed that they did not expect the future research to be as robust or “scientific” as the one required in the master’s degree, thus, they did not expect as many challenges.

However, three interviewees stated that although they understood the importance of research, their intention to conduct future research is low due to the negative experiences they had in the program. An IET participant addressed this contradiction by saying:

*I joined this program because I thought I could improve myself. In the end, I spend so much time fixing the (thesis) paper.*

This comment demonstrates that the so-called development of research competencies is manifested as requirements for thesis writing in the program. The institutional setting imposes assumptions regarding thesis writing as a master’s program requirement. In degree programs, teachers’ research is measured according to its conformity to academic writing conventions in terms of structure and knowledge of the field (Altrichter and Mayr, 2004). To earn a master’s degree and bolster their credentials to increase their competitiveness in the job market, teachers accept the restrictions and attempt to overcome the challenges involved in writing a thesis report.

Furthermore, the IETs who participated in this study were encouraged to apply innovative research ideas in a high-stakes examination-driven educational system, which creates conflict and challenges. In the comment given below, Jian explains his struggle while researching teaching English language speaking at Chinese high schools. Although teaching speaking is a potentially important topic, Jian says that he struggled with data collection and analysis, methodological limitations, and institutional requirements:

*Before I started the project, I thought Chinese high schools did not teach much English speaking because the education these English teachers received was outdated. I thought, “They [other English teachers] received English education twenty years ago, so they can't teach speaking.” But when I started to teach speaking, I found it was more complicated than that. I struggle a lot … College entrance examinations do not test speaking, so my mentor teacher keeps reminding me that I should not let “practice speaking” interfere with students' grades, so I got limited opportunities to try [it] out. [After I finally did my experiment on teaching speaking], I found that it is difficult to analyze speaking samples. I collected some students' speaking samples, but I found them difficult to grade and analyze. Maybe because I am not a native speaker, I am not proficient enough in the spoken language … Grading speaking is not like grading grammar. The grading standard for speaking [such as the IELTS speaking test] is very vague to me … it was such a struggle to write the report.*

Through his research experience, Jian realized that attempts to change English teaching practices need to consider schools’ examination-oriented culture. Jian’s job as a teacher is prescribed as improving students’ test results through repetitive practice, while as a researcher, he was expected to engage in reflection and possibly make positive changes in his teaching. His struggle demonstrates the gap between English language teaching practices in high schools and the theoretical understanding of English language teaching in academia which cannot be easily filled by a single teacher’s effort to change his teaching practice.

Since we found Jian’s research experience to be a particularly interesting case, we interviewed Jian’s advisor, seeking her opinion on Jian’s research project. She stated:

*If he had read the literature, he would have known that teaching speaking in Chinese high schools is difficult. I think speaking is an innovative topic for IETs, as we don't have many teacher researchers doing that, but it is a hard topic. He should have read the literature and been prepared for data analysis before he started the project, and we talked about this before he started data collection. Without adequate preparation, doing research on speaking is going to be hard.*

Jian’s advisor acknowledged that research on speaking is innovative but noted that before undertaking such a topic, Jian should have prepared by equipping himself with research skills such as data analysis. His academic advisor viewed his struggle as a knowledge-based one that could have been solved by adequate academic reading but failed to address the deeper cultural clashes that Jian experienced while problematizing the practices at his school (Taylor, 2017).

Although IETs are encouraged to modify and examine through their inquiries in the program, those participating in this study faced cultural, social, and institutional limitations on what research should encompass. Specifically, their schools expected them to focus on examination-driven practices, while their university emphasized innovation and robust academic reports. For Jian, such a clash between theory and reality could have served as the context for interesting research if he had focused on examining the nuances and complexities involved in the clash. Unfortunately, the program did not allow for this due to the positivist approach to research found in teacher education programs both within and outside China (Gao et al., 2011).

In addition to Jian, another IET interviewee, Hua, reported that the exam-oriented culture at her workplace makes the whole idea of teacher research irrelevant to their practices.

*In my school, many teachers joke that we are not teachers. We are tiger moms. We don't teach. We just push the students to practice and practice some more, and practice some more… so I chose a theoretical study that my advisor suggested [and approved]. It was good enrichment that deepened my knowledge of the English language.*

Hua reported that she perceived undertaking a theoretical project was useful for personal enrichment although due to a lack of agency in her teaching, the research might not help her change her practices. Instead of feeling empowered by teacher research, Hua’s agency was diminished, as she gave up on examining her teaching. In our analysis, suitable topics for Hua could have been “how to better motivate students” or reflecting on her lack of autonomy in teaching. When we proposed these options to Hua in the interview, she responded as follows:

*Those are definitely interesting topics. I did not know I could do that because no one around me had conducted research like that … I am a beginner teacher. No matter what I research, I don't feel like I can change the teaching practices at my school in any way. School principals and parents just want to see us teach like this [that is, using the approach we are using now].*

Thus, Hua’s case reveals that teacher research encompasses assumptions about teachers’ autonomy and agency in classrooms, which, in a highly examination-driven education system, are still weak (Guo et al., 2016). Hua was fully aware of the problems in her practices but she felt disempowered to change them due to the static assessment policies in China. In teaching contexts that do not promote teacher agency, meaningful teacher inquiry is nearly impossible even though the teacher was motivated to improve their practices through research (Taylor, 2017). Hence, attempts to understand teacher research attitudes must consider the larger socio-cultural and educational contexts in which teaching practices are embedded. Hua’s comments questioned the previous assumptions that positive attitudes toward research will lead to the use of research practices in future professional practices. Their schools tended to allow little space for experimentation and change. When the IETs had to grapple with the gap between the TESOL research and the realities of teaching in China. without adequate or appropriate guidance from their academic advisors and support from their school, connecting their practices with the broader knowledge of research is impossible.

## 4. Discussion

We presented a preliminary study to address the following central questions: For the IETs enrolled in a Chinese graduate degree program, what characterizes their attitudes toward research, quantitatively and qualitatively? What social and educational contexts promote or hinder their research efforts and their application of research findings in teaching practices from the IETs and their academic advisors’ perspective? This study adds new knowledge to the field of teacher education by presenting diverse IET and their advisors’ attitudes toward research and highlighting a need to reexamine the presumption that teachers’ positive attitudes toward research will transfer to a change of behavior in their careers.

The findings of the present study need to be interpreted in light of three major limitations. Firstly, this study is based on self-reported surveys and interviews. As with any self-reported survey, respondents often know what is expected of them and can therefore choose to answer in keeping with expectations rather than in a manner that reflects their authentic feelings and thoughts. However, even with the self-reported format, IETs reported diversified attitudes toward teacher research for various reasons, it is probable that the situation can be more diverse with other IETs. The second limitation is that this study only examined one graduate teacher education program for in-service teachers in China. Caution should be taken when generalizing the findings to other IET settings. Thirdly, this study used volunteers as participants. It is highly possible that our self-selected participants could be more motivated than average in their studies (as evidenced by their ability to finish the thesis), whose view could be skewed or biased in comparison to other potential participants who did not give consent to the study. One thing to note is that our findings suggested that even with this highly motivated group of IET, they still faced great challenges in their research endeavors. The situation can probably be worse with other IETs.

### 4.1. Chinese IETs’ research attitudes

To examine Chinese IETs’ research attitudes, this study adopts the reasoned action approach theory and uses triangulated data to examine dimensions of a pre-existing understanding of students’ research attitudes questionnaire. CFA of the seven dimensions of the RAVE-Q, based on van der Linden et al. (2015) and Griffioen (2019), suggests that six dimensions apply to IETs. However, the qualitative data shows that although some IETs expressed their positive attitudes toward research, teacher research encompasses assumptions about teachers’ autonomy and agency in classrooms, which are still weak in some Chinese schools.

The IET’s answers to the questionnaire are consistent with most of the teachers’ perceptions of research in degree programs as we described earlier. Most IETs perceived teacher research as useful opportunities and resources that have helped them develop inquiries and explore teaching with the benefit of new insights and reflection (e.g., Cochran-Smith et al., 2009; Guilbert et al., 2016; Yan, 2017; Byman et al., 2020). These findings extend insights from past research that mainly focus on pre-service teachers to include in-service teachers’ research experiences. Furthermore, we found that previous studies on pre-service teachers report their major outcome of research projects as to “learn how to become teachers” (van Katwijk et al., 2019), while in this project, comments on research outcome focused on “learn to reflect, change and innovate” as research inquiries were conducted by in-service teachers.

Such results show that although the teachers in this program are compelled to conduct research on their teaching, and that they had to battle the challenges such as lack of time to conduct research, with appropriate guidance, most of them appreciate the opportunities to conduct research. This is in line with Gao et al. (2011), Kowalczuk-Walędziak et al. (2019), and Yan and Yang (2019) studies.

### 4.2. Contextual conditions for teacher research

Despite their positive ratings on the intention to conduct research in the future, IETs expressed various challenges that needed to be conquered to conduct research in teaching. This finding is consistent with previous studies (Griffioen, 2019; van Katwijk et al., 2019), but the IETs encountered different challenges compared to some of their Western counterparts because of the contextual conditions.

The first challenge is a lack of agency for IETs in their classrooms. While some IETs were inspired to apply research-based pedagogy in their school, the examination-driven culture made the teachers’ efforts to change their teaching behavior impossible. When students, parents, and other stakeholders at school have a strong belief in the examination-driven culture, teachers feel disempowered rather than empowered by the research requirement, as other studies often assume (Cochran-Smith and Lytle, 2009). The second challenge is the disconnect between TESOL research and the stark realities faced by Chinese EFL teachers. Some IETs interviewed for the study reported that by applying research, they realized how disconnected research is from their reality rather than understanding the connection between research and reality (van Katwijk et al., 2019).

Finally, this study investigated faculty perspectives on IETs’ research attitudes, which have frequently been overlooked in prior studies (Yancovic-Allen, 2018). In the interviews, professors expressed their anticipation that IETs should read academically, think critically, and put more effort into their research. As previously noted, such expectations are set in contrast to what is expected of instructors in their teaching jobs.

Scholarship has investigated a variety of contextual issues relevant to teacher research (Zeichner, 2003; Kowalczuk-Walędziak et al., 2019; van Katwijk et al., 2019) specifically focusing on whether research is valued in schools and society. The Chinese context, however, proved to be unique to this topic: on the one hand, research is valued, and teachers are encouraged to conduct research for credentials and promotion; on the other hand, change of teaching practices according to empirical research evidence is not valued in classrooms. This study also raises a question about the practicality of EFL research. How little the current EFL studies do not take K-12 contexts in China into consideration (especially in rural areas) might explain the administrators’ lack of confidence in evidence-based research and the difficulties for teacher research.

### 4.3. Implications for encouraging teacher research in graduate EFL teacher education programs

This study has a few implications for encouraging teacher research in graduate EFL programs. First, in the Asia-Pacific context, teacher research should not be positioned as a magic wand for innovating teaching and bridging the gap between academia and practices (Borg, 2007). Instead, teacher education programs should aim to understand the complex realities that IETs face and provide guidance to help IETs negotiate their nuanced realities.

Secondly, the requirements for a master’s degree should not simply aim to gatekeep in response to academic degree inflation (Labaree, 1997), but also strive to provide students with an opportunity to engage in self-exploration and self-directed inquiry. Teacher research can be helpful when it is conducted with appropriate guidance from academic advisors, when students’ inquiry is self-directed, and when the school provides teachers with adequate support (Borg, 2007). However, programs that incorporate teacher research should encompass a wider range of methods and research paradigms, including autoethnography and reflective writing, that focus on individual teacher experiences. While writing for academia might empower teachers as knowledge creators (Cochran-Smith and Lytle, 2009), thesis writing as an institutional requirement in degree programs can also colonize teacher research and diminish teachers’ interest and their sense of power in research (Cheng and Li, 2020).

While the results and implications of this study might apply to other master’s teacher education programs in China as well as worldwide (Buschor and Kamm, 2015; Eklund, 2019), this study can be extended as follows: (1) This study is based on self-reported survey data and interviews conducted at one point in time. Future studies can administer multiple surveys throughout the program to understand IETs’ progress and their post-program situation. Future research should also examine the participants who could not finish their thesis studies in terms of the challenges that they experienced. (2) This study only includes English teachers. Further work could explore research experiences in different disciplines and determine whether subject matter affects teachers’ research experiences. (3) Fundamental issues concerning the program’s research culture will be important to examine to further understand teacher research in degree programs like this. While this program provides courses on topics such as second language acquisition, English language learning, and quantitative and qualitative research methods, there is a lack of understanding of how those courses conceptualize research. The IET advisor interviews in this study are preliminary as well. Future studies should examine faculty’s perspective on teacher research with more systematic surveys and interviews.

As Borg (2007) pointed out, the nature of the “research education” might be key in shaping the program participants’ attitudes toward research. These limitations need to be considered when applying the findings of this study.

## 5. Conclusion

In this study, we explored the research attitudes of a group of in-service English teachers enrolled in a master’s-level teacher education program in China. All the teachers in the study conducted research to fulfill the requirements for the graduate degree. Both quantitative and qualitative data show that, in general, IETs reported positive attitudes toward research: they understand the benefit and importance of teacher research as they perceived that teacher research contributed to their personal and pedagogical development, as well as to their understanding of scientific research.

However, the qualitative data also show that due to the high-stakes examination-oriented culture and the institutional requirements of the graduate degree, several IETs experienced substantial challenges and contradictions, as they were caught between their roles as teachers and researchers. While their graduate program encouraged them to conduct innovative research and question existing teaching practices, their schools tended to allow little space for experimentation and change. When the IETs had to grapple with this gap alone, that is, without adequate or appropriate guidance from their academic advisors and support from their school, they regarded their research experiences often as a hurdle to overcome to obtain a degree, instead of an enriching educational experience.

## Data availability statement

The raw data supporting the conclusions of this article will be made available by the authors, without undue reservation.

## Ethics statement

The studies involving humans were approved by the Shaanxi Normal University School of International Studies. The studies were conducted in accordance with local legislation and institutional requirements. The participants provided their written informed consent to participate in this study. Written informed consent was obtained from the individual(s) for the publication of any potentially identifiable images or data included in this article.

## Author contributions

LC was responsible for writing the manuscript. QL was responsible for constructing the ideas, data analysis, and data collection. All authors contributed to the article and approved the submitted version.

## Funding

This study is funded by Social Science Foundation of Shaanxi Province, 2020P027.

## Conflict of interest

The authors declare that the research was conducted in the absence of any commercial or financial relationships that could be construed as a potential conflict of interest.

## Publisher’s note

All claims expressed in this article are solely those of the authors and do not necessarily represent those of their affiliated organizations, or those of the publisher, the editors and the reviewers. Any product that may be evaluated in this article, or claim that may be made by its manufacturer, is not guaranteed or endorsed by the publisher.
